# Kawasaki disease with a concomitant primary Epstein - Barr virus infection

**DOI:** 10.1186/s12969-020-00459-0

**Published:** 2020-08-12

**Authors:** Nataly Rosenfeld, Diana Tasher, Adi Ovadia, Shirly Abiri, Ilan Dalal

**Affiliations:** 1 Department of Paediatrics, Edith Wolfson Medical Center, Halochamim 62, Holon, Israel; 2 Sackler Faculty of Medicine, Tel- Aviv University, Tel- Aviv, Israel

**Keywords:** Kawasaki disease, Epstein- Barr virus, Intravenous immunoglobulins

## Abstract

**Background:**

Kwasaki disease (KD) is the leading cause of acquired heart disease in children in most developed countries. The cause of KD remains unknown. The presumed theory is that KD occurs due to one or more infectious agents who evoke an abnormal immunological response in susceptible individuals. Epstein - Barr virus (EBV) infection has been considered as a suspected causative agent because of the potential effect on the immune system.

**Case presentation:**

A previously healthy 19 month old boy presented with a 6 day history of fever accompanied by a diffuse macular erythematous rash that appeared 1 day after. The physical examination on admission revealed bilateral non-suppurative conjunctivitis, dry fissured and injected lips without “strawberry” tongue, diffuse macular rash on the trunk, face and limbs, swelling of the hands and feet, and right cervical lymphadenopathy (2 cm in diameter). Following fulfillment of all the clinical criteria, the diagnosis of KD was made and treatment with IVIG 2 g/Kg was administered along with oral aspirin (80 mg/ kg/day). However, despite the treatment, he remained febrile for an additional 2 days with persistent clinical manifestations. Therefore, he received a second 2 g/kg IVIG course with a favorable response. On the 14th day of illness the patient became febrile again and was readmitted. Blood examinations revealed remarkable leukocytosis up to 35.7 X 10^9^/L with 87.3% lymphocytes and the blood smear revealed atypical lymphocytes and monocytes. The liver enzymes were elevated. The serology for infectious mononucleosis from his first admission revealed: IgM CMV (+), IgG CMV (−); IgM VCA EBV (+) IgG VCA EBV (−), IgG EBNA (−). To confirm infectious mononucleosis following the administration of 2 doses of IVIG, serum EBV PCR was performed and was positive (1.6X 10^3^ cp/ml).

**Conclusions:**

We describe here a case of KD with a concomitant primary EBV infection. To the best of our knowledge, this is the first case in western country that describes KD with acute EBV infection as confirmed by PCR. The case we described stands as a contribution in favor of the possible role of EBV in the development of KD.

## Background

Kawasaki disease (KD), also known as acute febrile mucocutaneous lymph node syndrome (MCLS), is an acute febrile illness of childhood seen worldwide with the highest incidence occurring in Asian children. KD is a vasculitis with a predilection for the coronary arteries. The treatment of choice is Intravenous immunoglobulins (IVIG) and aspirin. Twenty-five percent of untreated children develop coronary artery abnormalities (CAA) including aneurysms, whereas less than 5% of children treated with IVIG develop CAA. Approximately 15 to 20% of treated children require a second dose of IVIG [1]. KD is the leading cause of acquired heart disease in children in most developed countries. The cause of KD remains unknown. The presumed theory is that KD occurs due to one or more infectious agents that evoke an abnormal immunological response in a susceptible individual [2, 3]. Epstein - Barr virus infection (EBV) in particular has been considered as a suspected causative agent because of its potential effect on the immune system. EBV infection is associated with immunological disorders such as hemophagocytic syndrome, lymphoproliferative disorders and Burkitt’s lymphoma.

We describe a case of a 19 month-old male infant who developed KD with a concomitant primary EBV infection.

## Case presentation

A previously healthy 19 month old boy presented with a 6 day history of fever accompanied by a diffuse macular erythematous rash that appeared 1 day after. The physical examination on admission revealed bilateral non-suppurative conjunctivitis, dry fissured and injected lips without “strawberry” tongue, diffuse macular rash on the trunk, face and limbs, swelling of the hands and feet, and right cervical lymphadenopathy (2 cm in diameter). Investigations revealed a total white blood count of 21 X 10^9^ /L (neutrophils: 27.5%, lymphocytes: 58.4%, monocytes: 12.6%), hemoglobin: 11.9 g/dL, platelet count: 171X10^9^/L. The peripheral blood smear revealed atypical lymphocytes. Laboratory investigations also showed elevated C-reactive protein (CRP) 8.5 mg/dl (normal reference ≤0.5 mg/dl), hypoalbuminemia (3 g/dl) and elevated transaminase levels: alanine aminotransferase (ALT) of 132 U/L, alkaline phosphatase (ALP) of 375 U/L. Urinalysis was normal. Chest X-ray revealed peri-bronchial thickening without infiltrate. Serology for EBV and Cytomegalovirus (CMV) was ordered. Because of high suspicion of KD, an abdominal ultrasonography and echocardiography were performed. Abdominal ultrasonography revealed hydrops of the gallbladder with patent biliary tracts, enlarged porta hepatis lymph nodes up to 8.4 mm in diameter and enlarged spleen 3.5X10.5 cm in diameter. Doppler Echocardiography was normal without any evidence of CAA. Following fulfillment of all the clinical criteria, the diagnosis of KD was made and treatment with IVIG 2 g/Kg was administered along with oral aspirin (80 mg/ kg/day). However, despite the treatment, he remained febrile for an additional 2 days with persistent clinical manifestations such as conjunctivitis, red fissured lips, “strawberry tongue”, rash and peripheral edema; therefore he received a second 2 g/kg IVIG course with a favorable response. Repeated echocardiography on the fifth day from admission was normal. The patient was discharged after resolution of fever and improvement of inflammatory markers. The blood tests on discharge revealed CRP of 2.2 mg/dl, albumin of 2.93 g/dl, ALT of 258 U/L, ALP of 618 U/L, Gamma-Glutamyl Transferase (GGT) of 497 U/L. He remained afebrile for only 2 days. On the 14 day of illness the patient became febrile again and was readmitted. The physical examination revealed periungual desquamation of the fingertips and toes without the typical stigmata of KD he had prior. Blood examinations revealed remarkable leukocytosis up to 35.7 X 10^9^/L with 87.3% lymphocytes, hemoglobin of 11.2 g/L, platelets of 233X10^9^/L and CRP of 1.8 mg/dl. The blood smear revealed atypical lymphocytes and monocytes. The liver enzymes were still elevated, ferritin was 405 ng/ml (normal reference 7–142 ng/ml) and triglycerides were 223 mg/dl. The serology for infectious mononucleosis from his first admission revealed: IgM CMV (+), IgG CMV (−); IgM VCA EBV (+) IgG VCA EBV (−), IgG EBNA (−). Based on the clinical and laboratory manifestations, we conclude that the fever was attributed to the EBV infection rather than KD relapse and therefore he was not treated with a third dose of IVIG. To confirm infectious mononucleosis following the administration of 2 doses of IVIG, serum EBV PCR was performed and was positive (1.6X 10^3^ cp/ml). Urinary CMV PCR was negative. The fever persisted for an additional 7 days. The patient was discharged with a low dose of aspirin. During the 3 months of follow-up, the patient remained completely asymptomatic and repeated echocardiograms were normal.

## Discussion

We describe here a case of KD with a concomitant primary EBV infection. To the best of our knowledge, this is the first case in western country that describes KD with acute EBV infection as confirmed by PCR.

The epidemiology of KD suggests infectious origin: endemic disease with epidemics, self-limited nature and seasonal predominance. Over 80% of cases occur between the ages of 6 months and 4 years. The low incidence of KD in the first 6 month suggests immunity by maternal antibodies [3]. The infectious agents that have been investigated include bacteria such as *Propionibacterium*, *Staphylococcus aureus*, *Streptococcus pyogenes* and *Chlamydia* as well as viruses such as EBV, Parvovirus, Retrovirus etc. [2, 3]. The genetic role in the pathogenesis of KD is emphasized by the fact that there is a higher risk in Northern- Eastern Asian children and in siblings and children of individuals with a previous history of KD [3]. The acceptable theory is that infectious agents cause an inappropriate immunological response to antigen or even superantigen in genetically susceptible individuals [4]. Timothy et al. studied coronary artery aneurysms from eight fatal acute KD by means of immune-histochemical studies [5]. They found that all of the biopsies demonstrated marked transmural infiltration by CD45RO^+^ T lymphocytes. Moreover, there was more than fourfold increase in the numbers of CD8 T lymphocytes in the inflammatory infiltrates compared with CD4 T lymphocytes. This article strongly suggests antigen driven immune response involving CD8 T lymphocytes and major histocompatibility complex class **I** which is suitable for intracellular pathogen infection, such as viruses. The leading assumption is that KD arises from common pathological processes, although the antigenic triggers and the genetic determinants may differ between populations. The role of EBV infection in the pathogenesis of KD remains elusive. Kikuta et al. previously reported the results of serological studies of EBV in patients with KD. Forty- nine (86%) of 57 KD and 15 (68%) of 22 patients with recurrent KD had serological evidence of primary EBV infection during the first month after the onset of KD [6]. Another report detected EBV PCR sequences in peripheral blood mononuclear cells in 21 (60%) of 35 KD patients within 2 weeks after the onset of KD. Furthermore, EBV sequences were detected within 3 months after the onset of KD in 6 patients. In comparison, only 2 (12%) out of 17 control DNA samples were EBV positive [7]. In contrast, Shigeto et al. found that EBV sero-positivity rates of KD patients aged 1–6 years were significantly lower than the corresponding age group. He claimed that EBV infection may have a defensive factor for the onset of KD. The suggested defensive mechanism is that Epstein-Barr viral proteins may modulate the host -organism interaction [8].

EBV serology is the most common technique to confirm EBV infection in immunocompetent patients [6, 8, 9]. Since both EBV VCA IgM and CMV IgM were positive, it was not possible to confirm EBV infection or CMV infection, due to the possibility of false positive results [10]. Moreover, the use of serology for EBV infection after treating with IVIG, which is the treatment of choice in KD, can cause false positive or negative results.

In this patient, the diagnosis of concomitant EBV infection was supported by positive serology for acute EBV infection (positive IgM VCA EBV antibodies), lymphocytosis with atypical lymphocytes on peripheral blood smear, elevated liver enzymes and confirmed by positive PCR. Based on the clinical presentation, we presume that EBV infection occurred first and that KD developed in an overlapping manner. We believe that the resolution of fever paralleled the recovery from KD while recurrence of fever after IVIG treatment may be attributed to the concurrent EBV infection. However, it remains debatable if the case represents a real KD associated with EBV infection, or it is simply an EBV infection with some clinical features resembling KD as the patient did not show coronary involvement, which would have made the diagnosis of KD indisputable.

## Conclusion

The case we described stands as a contribution in favor of the possible role of EBV in the development of KD. Further investigations with larger scale populations need to be done using PCR, flow cytometry and new immunological techniques to finally understand the unique immunological and genetic interaction between KD and EBV infection.

## Data Availability

The datasets during and/or analyzed during the current study available from the corresponding author on reasonable request.

## Abbreviations

KD: Kawasaki Disease
MCLS: Mucocutaneous Lymph Node Syndrome
IVIG: Intravenous Immunoglobulins
CAA: Coronary Artery Abnormalities
EBV: Epstein- Barr Virus
CMV: Cytomegalovirus
CRP: C-Reactive Protein
ALT: Alanine Aminotransferase
ALP: Alkaline Phosphatase
GGT: Gamma-Glutamyl Transferase
